# Mortality of surgically treated 80-year-old or older intracranial meningioma patients in comparison to matched general population

**DOI:** 10.1038/s41598-021-90842-y

**Published:** 2021-06-01

**Authors:** Ilari Rautalin, Christoph Schwartz, Mika Niemelä, Miikka Korja

**Affiliations:** 1 Department of Neurosurgery, University of Helsinki and Helsinki University Hospital, P.O. Box 266, 00029 Helsinki, Finland; 2 Department of Neurosurgery, University Hospital Salzburg, Paracelsus Medical University, Salzburg, Austria

**Keywords:** CNS cancer, CNS cancer, Surgical oncology

## Abstract

Population aging is likely increasing the number of surgically treated very old (≥ 80–year-old) intracranial meningioma (IM) patients. Since there is little data on mortality in this patient group, we studied whether survival of surgically treated very old IM patients differs from survival of a matched general population. We retrospectively identified 83 consecutive very old IM patients (median age 83 years; 69% women) operated between 2010 and 2018. During the first postoperative year, operated IM patients suffered 2.5 times higher mortality as compared to age- and sex-matched general population but no annual survival difference occurred thereafter. Regarding cumulative estimates, no excess mortality was detected after the second postoperative year. Of the patient who were and who were not able to live at home preoperatively, 78% and 42% lived at home within 3 months, respectively. Preoperative loss of capability to live at home associated with a less frequent return to home [odds ratio (95% confidence interval) 0.21 (0.06–0.67)]. Operated very old IM patients had short-term excess mortality but similar cumulative survival as the matched general population. Moreover, most patients returned home soon after surgery.

## Introduction

The number of very old (≥ 80-year-old) yet independent intracranial meningioma (IM) patients is constantly increasing as populations age^1^. However, tumor-related symptoms, such as motor deficits, seizures and impaired cognition, may quickly end an independent life and perhaps even life in general in this fragile patient group^2^. A very old age increases the risk of adverse surgical outcome, but carefully selected IM patients can sustain or even improve their functional status following the surgery^3–8^. Nevertheless, data on the surgical outcome of very old IM patients is limited^9^, and no studies to date have addressed excess mortality or home return after surgery in this patient group.

We retrospectively studied surgical outcomes of very old IM patients who were operated in a high-volume university hospital. We focused particularly on postoperative excess mortality and on postoperative capability to live at home (CLH). Our primary hypothesis was that very old IM patients suffer from postoperative excess mortality in comparison to the matched general population. The secondary hypothesis was that preoperatively dependent very old IM patients do not recover their independence and return to home after surgery. In other words, we hypothesized that major cranial surgery is a high-risk procedure in very old IM patients, and thus unlikely to be associated with favorable outcome.

## Methods

### Ethical considerations

The local institutional review board of Helsinki University Hospital (HUH) approved the data extraction from the electronic medical record systems of the study hospital, and granted a waiver of consent for this retrospective chart review study. The study followed the STROBE (Strengthening the Reporting of Observational Studies in Epidemiology) checklist and ethical principles of the Declaration of Helsinki^10^.

### Study hospital

All included patients were operated by one of the twelve neurosurgeons of the Department of Neurosurgery, HUH. HUH has a catchment area of approximately 2.2 million inhabitants, and performs more than 90,000 surgeries every year. The Department of Neurosurgery, which is the largest neurosurgical unit in Finland, performs nearly 4000 annual operations. All five Finnish university hospitals are publicly funded non-profit organizations that provide tertiary health care services for Finnish inhabitants.

### Patient identification

The study cohort has been described previously^8,11,12^. By utilizing a hospital-based electronic Centricity™ Opera (GE Healthcare) software, which is an operating theatre management solution, we identified all very old (≥ 80-year-old) patients who underwent their first-ever elective IM surgery at HUH. The register has been in use since mid-2009, hence we included patients who were operated on between January 1, 2010 and December 31, 2018. To identify all eligible meningioma patients, we manually reviewed the medical notes of the identified patients through the electronic medical record (EMR) system (Uranus™, CGI). All patients with non-meningeal tumors or previous IM operations were excluded.

### Risk scales

We used the previously validated Helsinki version of the American Society of Anesthesiologist (Helsinki ASA) scale to assess operative risks of included patients^13^. The Helsinki ASA score has been designed to provide preoperative stratification specifically for elective craniotomy patients^13^, and has been in clinical use in the Neurosurgical Department of HUH since the mid 1990s. In addition, based on medical notes in the EMR system, we estimated preoperative Karnofsky Performance Status (KPS)^14^ scores for every patient.

### Patient and tumor characteristics

In addition to age and sex, we collected information about preoperative symptoms and surgical indications. In terms of tumor characteristics, we defined the maximum diameter, location, and multiplicity of IMs by using magnetic resonance images. Specifically, we measured the maximum diameter in three anatomical planes (coronal, sagittal and axial), and used the largest diameter (excluding the dural tail) to describe the size of IM. Tumors with a maximum diameter of ≤ 5 cm were considered small^9^. For location, we used five categories: 1) convexity, 2) falx, 3) supratentorial skull-base, 4) posterior fossa, and 5) other locations^3–7^. We also reviewed pathology reports, and recorded the WHO grade (I–III)^15^ of IMs. Surgery time (skin-to-skin in minutes) and operation code defining the extent of resection (partial/total) were extracted from the Centricity™ Opera for every patient.

### Follow-up

To determine the IM patients’ postoperative performance and CLH, we reviewed the medical records up to 1 year after surgery. In terms of survival, the nationwide patient data repository (the Population Register Center) provided the information of any deaths during the follow-up. The follow-up started at the time of surgery and ended on the 13^th^ of September 2020 or to death, whichever came first.

### Outcome measures

In addition to excess mortality, we assessed in-hospital, 30-day and 1-year mortality rates. From the medical notes of the EMR system, we recorded whether IM patients were capable to live at home (with or without home care services) preoperatively, at discharge and at 3 months after the surgery. In Finland, CLH is routinely recorded in the medical notes, specifically for very old patients. Moreover, we collected information about the length of hospitalization (days), as well as about postoperative complications. Due to the retrospective design, we focused on major complications^16^, which are more reliably reported in the medical notes. We evaluated performance status changes by estimating the KPS scores from the medical notes preoperatively, at discharge and in the last follow-up visit within the first postoperative year.

### Statistical analyses

For excess mortality calculations, we compared observed survival rates to age-, sex- and year-matched survival rates in the general Finnish population. We obtained this general population data from a public website of Statistics Finland (https://pxnet2.stat.fi/PXWeb/pxweb/en/StatFin/). We calculated yearly estimates for both year-specific and cumulative survival rates using the Ederer II survival method^17^. Using a multiple logistic regression model, we evaluated if the previously reported risk factors for adverse outcome^3–7,18,19^ were also associated with postoperative mortality or ability to live at home after surgery in our cohort. Since the main objective of our multivariable analyses was to identify factors that may associate with (not cause) adverse outcome in this fragile patient cohort, we adjusted the multivariable models using the significant factors found in univariable models^20^. In the regression analyses, we dichotomized categorical variables, such as Helsinki ASA scale (< 4 = mild/none or ≥ 4 significant tumor-related symptoms) and tumor location (skull base or other location). Odds ratios (ORs) with 95% confidence intervals (CIs) described the associations when applicable. As post hoc analyses, we also calculated excess hazard ratios (EHRs) for age, sex and preoperative loss of CLH to investigate whether these factors also contribute to the observed 1-year excess mortality. As recommended^17^, we included only categorical variables in this adjusted multivariable model, and thus dichotomized age by its median value. We used Stata version 16.1 (Stata Corp, College Station, TX) for all statistical analyses.

## Results

### Patient and tumor characteristics

Patient and tumor characteristics are described in Table 1. Between 2010 and 2018, 83 IM patients underwent the first-ever IM surgery at the age of 80 years or older. The median age of operated patients was 83 years (range 80 to 96 years), and most of them (69%) were women. Prior to surgery, nearly half (41%) of the patients presented with significant tumor-related symptoms (Helsinki ASA class 4), which in turn had led to the loss of CLH in 30% of the whole cohort. Only one patient lived in a health-care institution prior to the onset of tumor-related symptoms. The three most common surgical indications that were listed in the medical records were cognitive decline/psychomotor changes, motor deficits, and visual impairment. In terms of tumor characteristics, IMs were located most often in the skull base (40%), and were small (66%). The majority were graded as 1 (73%) according to the WHO grading system, and were resected completely (93%). Multiple meningiomas were found in 10% of the patients (Table 1).

**Table 1:** Patient and tumor characteristics.

Patient characteristic	
N of cases (%)	83 (100)
**Age, median (range)**	83 (80–96)
**Female sex, n (%)**	57 (68.7)
**Helsinki ASA class, n (%)**	
I	0 (0)
II	5 (6.0)
III	44 (53.0)
IV	34 (41.0)
V	0 (0)
**KPS before onset of IM-related symptoms, median (IQR)**	80 (70–90)
**Preoperative KPS, median (IQR)**	60 (40–70)
**Surgical indications, n (%)**	
Cognitive impairment	30 (36.1)
Hemiparesis/motor deficit	18 (21.7)
Visual loss	9 (10.8)
Balance disturbance	6 (7.2)
Seizure	5 (6.0)
Asymptomatic tumor growth	5 (6.0)
Gait impairment	4 (4.8)
Aphasia	2 (2.4)
Other (headache, dermal effusion, hydrocephalus)	3 (3.7)
Missing	1 (1.2)
**Surgical time (min), median (IQR)**	148.8 (117.0–217.2)
**Length of hospitalization (days), median (IQR)**	7 (5–8)

Tumor characteristic	
I	52 (62.7)
II	19 (22.9)
III	0 (0)
Missing	12 (14.5)
**IM maximum diameter, n (%)**	
≤ 5 cm	55 (66.3)
> 5 cm	28 (33.7)
**IM location, n (%)**	
Convexity	31 (37.4)
Falx	9 (10.8)
Skull-base	33 (39.8)
Posterior fossa	7 (8.4)
Other	3 (3.6)
**Multiple meningioma, n (%)**	8 (9.6)
**Extent of resection, n (%)**	
Partial	6 (7.2)
Total	77 (92.8)

*ASA* American Society of Anesthesiologist; *IM* intracranial meningioma; *IQR* interquartile range; *KPS* Karnofsky Performance Status; *WHO* World Health Organization.

### Complications

Over one-fourth (27%) of the operated IM patients had one or more major postoperative complications. The most common major complications were an intracranial hemorrhage (causing mass effect and/or requiring reoperation, n = 8), new epileptic seizure (n = 5) and pneumonia (n = 5) (Supplementary Table 1). An increasing age was the only factor associating with a higher risk of major complications (OR 1.32 (1.08–1.61) per each year-increase in age) (Supplementary Table 2).

### Mortality

Two IM patients (2%) died in the hospital; one due to a major intracranial hemorrhage (did not wake up after the surgery) and one due to a sudden cardiac arrest 4 days after surgery. Thirty-day and 1-year mortality rates were 7% and 18%, respectively. Overall, 33 (40%) out of 83 patients died during the median follow-up of 4 years (range 2 days to 11 years). According to the adjusted regression model, each year-increase in age associated with the increased risk of both 30-day (OR 1.36 (1.05–1.75)) and 1-year mortality (OR 1.91 (1.27–2.89)). Moreover, the preoperative loss of CLH was associated with a higher 1-year mortality risk (OR 5.28 (1.11–25.26)) (Table 2). In addition, the Helsinki ASA score of 4 and each 10-unit decrease in the preoperative KPS were associated with increased 30-day and 1-year mortality rates in the univariable analyses, but were not significant in the multivariable model (Table 2). Mortality rates were similar in men and women.

**Table 2:** Odds ratios (ORs) with 95% confidence intervals (95% CIs) for short-term (30-day) and 1-year mortalities. Multivariable model includes significant factors from univariable model and patients’ sex.

Variables	30-day mortality		1-year mortality	
	Univariable	Multivariable	Univariable	Multivariable
**Age (per each year)**	1.46 (1.12–1.91)	1.36 (1.05–1.75)	1.79 (1.29–2.49)	1.91 (1.27–2.89)
**Sex**				
Male	(Reference)	(Reference)	(Reference)	(Reference)
Female	0.43 (0.08–2.27)	0.41 (0.05–3.34)	0.63 (0.20–1.99)	0.51 (0.10–2.52)
**Helsinki ASA score**				
2–3	(Reference)	NA	(Reference)	(Reference)
4	8.28 (0.92–74.39)	NA	3.67 (1.12–11.97)	2.25 (0.48–10.43)
**Preoperative KPS (per 10-unit increase)**	0.48 (0.25–0.93)	0.51 (0.24–1.06)	0.61 (0.40–0.90)	NA*
**Preoperative capability to live at home**				
Yes	(Reference)	NA	(Reference)	(Reference)
No	5.00 (0.85–29.29)	NA	4.50 (1.40–14.50)	5.28 (1.11–25.26)
**Tumor size (per one cm increase)**	1.13 (0.64–1.99)	NA	1.07 (0.73–1.57)	NA
**Tumor location**				
Other	(Reference)	NA	(Reference)	NA
Skull-base	0.74 (0.13–4.30)	NA	0.71 (0.22–2.32)	NA
**Surgical time (per 30 min increase)**	0.84 (0.56–1.27)	NA	0.73 (0.53–1.01)	NA
**Extent of resection**				
Partial	(Omitted)	NA	(Reference)	NA
Total	(Omitted)	NA	1.11 (0.12–10.27)	NA

*ASA* American Society of Anesthesiologist; *KPS* Karnofsky performance status; *NA* not applicable.

*Due to strong correlation between preoperative KPS and preoperative independence, we excluded preoperative KPS from the multivariable model for 1-year mortality.

### Excess mortality

**Figure 1:**
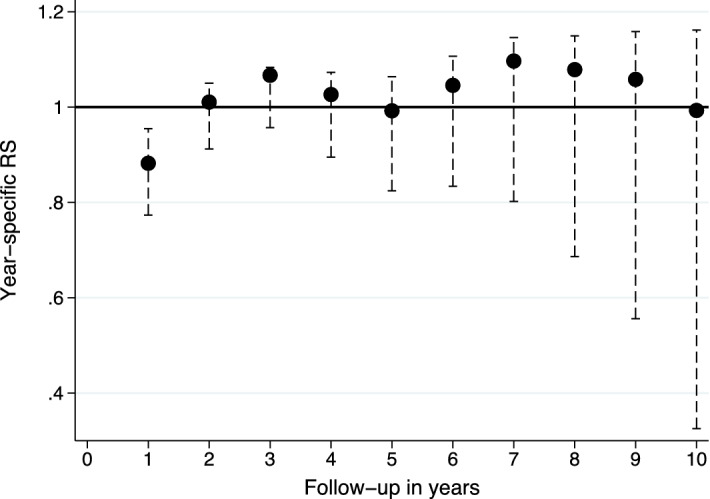
Year-specific relative survival (RS) (dots) with 95% confidence intervals (whiskers).

**Figure 2:**
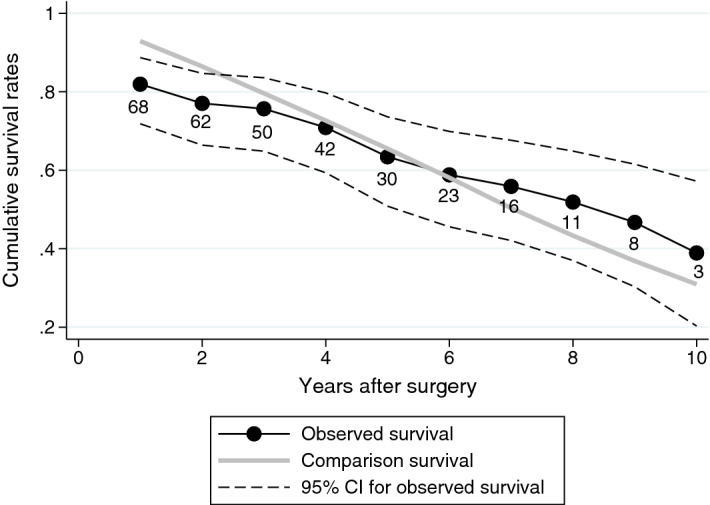
Cumulative survival rates of operated IM patients (black connected line) with 95% CIs (black dashed lines) and the age-, sex- and year-matched general Finnish population (grey solid line). Difference between solid lines depicts the relative survival, whereas marked values below the observed survival line (black connected line) describe the number of patients whose follow-up lasted at least until the yearly time point.

As compared to the age- and sex-matched general population, very old IM patients had an excess mortality of 150% during the first postoperative year. However, the survival rates were similar thereafter (Fig. 1). In terms of cumulative estimates, excess mortality levelled off from the third postoperative year onwards (Fig. 2). During the first postoperative year, the age over the median value (83 years) was associated with an increased excess mortality (EHR = 7.98 (1.18–53.72)). Similarly, the preoperative loss of CLH associated with the increased risk of excess mortality (EHR = 10.13 (1.38–74.30)) compared to the patients who were able to live at home prior to surgery. There were no differences between men and women.

### KPS change

**Figure 3:**
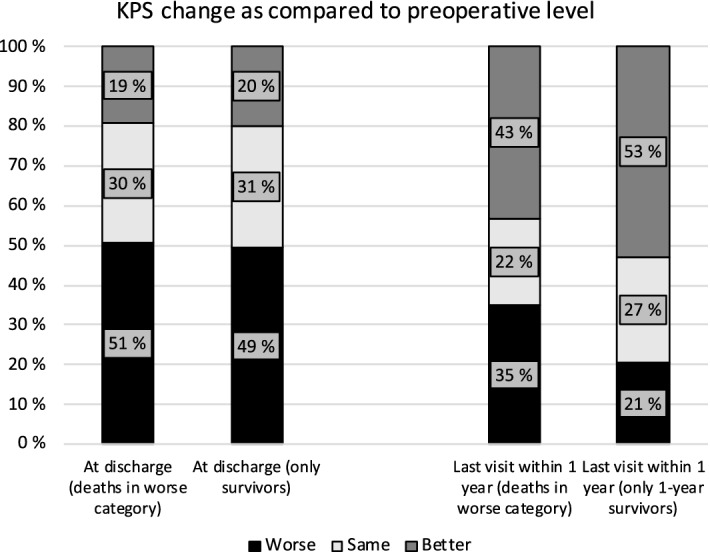
Comparison between pre- and postoperative performance levels (measured by KPS) at discharge and at the last follow-up visit within the first postoperative year.

At discharge, half (51%) of the patients had lower KPS values than preoperatively. However, the majority of operated IM patients (65%) recovered fast and reached the same or improved performance level during the first postoperative year (Fig. 3). When evaluating only the patients who were alive after the first year, 53% improved and 26% sustained their performance levels (Fig. 3).

### Capability to live at home (CLH)

**Figure 4:**
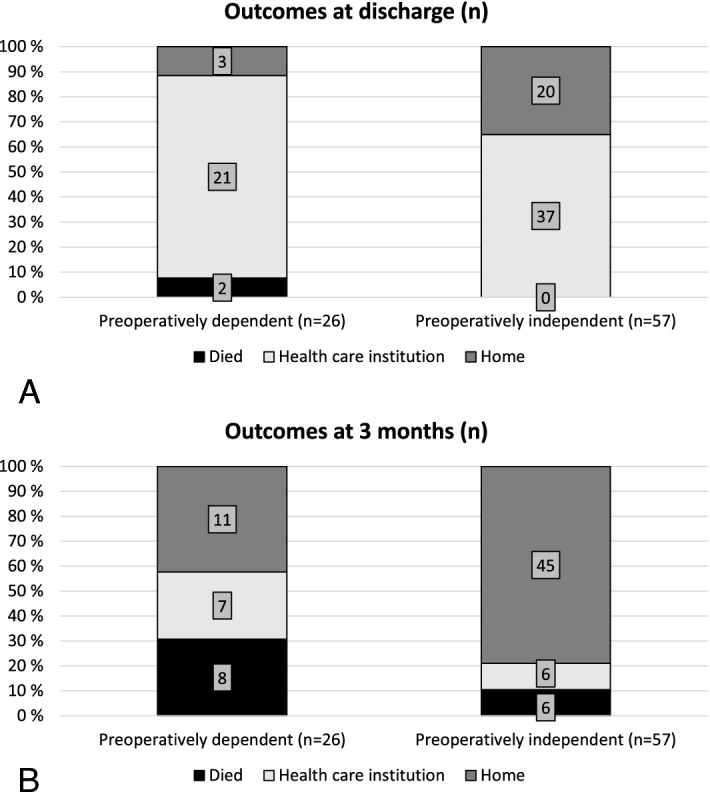
Postoperative capability to live at home (A) at discharge and (**B**) at 3 months. Preoperatively independent patients illustrate the patient who were able to live at home before surgery whereas preoperatively dependent patients illustrate patients who had lost their capability to live at home preoperatively.

Of the 26 patients who were not capable to live at home prior to surgery, 2 (8%) died in hospital, and 3 (12%) regained their independence and returned home at discharge. Of the patients who lived at home prior to surgery (n = 57), 37 (65%) required institutional rehabilitation after surgery (Fig. 4). At 3 months, 11 (42%) of the patients that lost their CLH preoperatively and 45 (79%) of the patients that had CLH preoperatively were living at home (Fig. 4). In the adjusted multivariable model, each year-increase in age (OR 0.71 (0.55–0.92)) and the preoperative loss of CLH (OR 0.20 (0.06–0.63)) were associated inversely with the ability to live at home after 3 months (Table 3).

**Table 3:** Odds ratios (ORs) with 95% confidence intervals (95% CIs) for 3-month independence. Multivariable model includes significant factors from univariable model and patients’ sex.

Variables	3-month capability to live at home, OR (95% CI)	
	Univariable	Multivariable
**Age (per each year)**	0.73 (0.59–0.90)	0.71 (0.55–0.92)
**Sex**		
Male	(Reference)	(Reference)
Female	1.47 (0.56–3.89)	1.64 (0.52–5.20)
**Helsinki ASA score**		
2–3	(Reference)	(Reference)
4	0.33 (0.13–0.84)	0.48 (0.16–1.47)
**Preoperative KPS (per 10-unit increase)**	1.90 (1.31–2.73)	NA*
**Loss of preoperative independence**		
No	(Reference)	(Reference)
Yes	0.20 (0.07–0.53)	0.20 (0.06–0.63)
**Tumor size (per one cm increase)**	0.99 (0.72–1.36)	NA
**Tumor location**		
Other	(Reference)	NA
Skull-base	0.48 (0.19–1.22)	NA
	1.05 (0.88–1.25)	NA
**Extent of resection**		
Partial	(Reference)	NA
Total	0.39 (0.04–3.53)	NA

*ASA* American Society of Anesthesiologist; *KPS* Karnofsky Performance Status; *NA* not applicable.

*Due to strong correlation between preoperative KPS and preoperative independence, we excluded preoperative KPS from multivariable model.

## Discussion

In our series of 83 very old consecutively operated IM patients, we found that patients suffered from a significant short-term excess mortality in comparison to the matched general Finnish population. Based on the difference between observed (15/83) and expected (6/83) deaths in the first year, surgery led to 9 additional (2.5 times higher number) deaths. Most (60%) of the patients who died within the first year had lost their CLH prior to surgery because of the meningioma-related symptoms, so it is likely that the 1-year mortality rate of these symptomatic patients would have been relatively high even without surgery. Therefore, the 1-year excess mortality may be an overestimate, and in fact the mortality rate should be compared to age- and sex-matched meningioma patients who were conservatively treated. However, such a prospective trial might be unethical to conduct. Following the second postoperative year, the survival rate and cumulative excess mortality rates of the operated patients were similar to the general population. Even though the risk of death was increased for the patients who were not able to live at home preoperatively, almost half of these patients returned home during the first three postoperative months. Of the patients who were able to live at home before surgery, 75% were also able to return home within the first 3 months. Based on the presented results, a careful patient selection seems to lead to a fair outcome.

Increased age and the preoperative loss of CLH were associated with the inability to live at home after surgery. Since increased age and the preoperative loss of CLH were relatively independent factors that were also associated with 1-year mortality, we conducted post hoc analyses in order to further elucidate if these two factors also contributed to excess mortality. Indeed, age over the median value of 83 years was associated with excess mortality. In addition, the patients who lost their CLH preoperatively had higher excess mortality. Given that the life expectancy of very old and institutionalized patients is shortened^21,22^, our findings suggest that if surgical treatment is considered as a reasonable option for very old IM patients, surgery should perhaps be performed when patients are still in good condition and physically active rather than wait until progressive symptoms cause major functional deterioration and immobilization. It is important to note that a long-term (i.e. several weeks) high-dose corticosteroid treatment may slow the symptoms down, but the treatment may be complicated by myopathy, particularly in elderly patients^23^. We have seen a few very old IM patients with severe corticosteroid treatment-related myopathy, and such an adverse condition may further complicate the surgery and recovery.

Our results are in line with previous findings^13,19,24,25^ that have suggested that older age is related to high complication and mortality rates, and dependence after IM resection or neurosurgery in general. Of note, from five oldest patients (age range 88–96) in our series, four suffered from major postoperative complications and died during the first 3 months. In addition, the fifth patient was not able to live at home after the surgery. Overall, over one-fourth of the patients suffered from major postoperative complications. Based on a prospective and comprehensive complication study, this rate is higher than the overall complication rate of cranial surgeries (27% vs. 18%; difference of 7 patients in our series)^16^, which underlines the increased risk of neurosurgery in older patients. On the other hand, in-hospital mortality rate among the very old IM patients was similar to the figure of the same prospective cohort^16^ (2% vs 1%; difference of only 1 patient in our series). Therefore, we believe that the overall results are still rather acceptable. In addition to a selection bias, relatively short surgical skin-to-skin times as well as the short median hospital stay may contribute to the recovery rate and subsequently to the overall results. Only one of the previous studies^4^ of very old IM patients reported the mean surgery time, which was 73 min higher than in our series (249 min vs. 176 min). Similarly, only one previous study^3^ reported the mean length of hospitalization, which was 12 days longer than ours (19 days vs. 7 days). Institutional care is known to be associated with increased mortality and morbidity, as well as with increased healthcare expenses^22^. Of the 58 patients discharged to institutional rehabilitation, over half (59%) returned home within three postoperative months, which may be related to the shorter length of hospitalization.

In addition to the current study evaluating the overall rationality of operating very old meningioma patients, we have previously used the same patient cohort to assess the effect of peritumoral edema^8^, size^12^ and surgeon’s experience^11^ on surgical outcomes. Based on our studies, surgical removal of IMs with larger peritumoral edema associates with more favorable outcome^8^, whereas surgical treatment of giant (diameter ≥ 5 cm) IMs entails a high complication rate^12^. Moreover, surgical results did not depend on the surgeon’s experience^11^. Besides our own studies, to the best of our knowledge, eight studies^3–7,18,19,26^ have assessed the surgical outcomes of very old (80 years or older) IM patients. However, no previous study has assessed excess mortality or patients’ postoperative return to home. In terms of survival, short-term (within 30 days) mortality rates have varied between 0 and 29%^3–7,18,19,26^, and 1-year rates between 9 and 29%^3,5,6,18^. The largest previous hospital-based study (n = 74) by Sacko et al.^3^ reported lower 30-day (0% vs. 7%) and 1-year mortality rates (9% vs 19%) than in our study. Noteworthy differences between study cohorts include the mean age, which was somewhat younger in their series than in ours (82 years vs. 83.3 years), and the location of the meningiomas, which were located less often in the skull base area in their series (16% vs 40%). Moreover, no surgeries were performed for patients older than 90 years. In terms of preoperative functional status, the proportion of patients with KPS ≥ 60 (57%) was similar to ours (62%). In the second largest hospital-based study (n = 51), Konglund et al.^18^ also reported relatively low 30-day (4%) and 1-year mortality rates (16%), but their operated patients were in notably better condition at the time of surgery (KPS ≥ 80 in 41% of their patients, compared to 14% of our patients). Moreover, they did not operate on patients older than 90 years.

Our study may have some strengths. First, we assessed year-specific and cumulative excess mortality rates for the first time, providing an accurate estimation of any survival disadvantages or benefits in very old IM patients. Second, we assessed—also for the first time—IM patients’ capability to return home after surgery. Given that this measure can be easily determined in Finland, even using a retrospective medical data, we believe it serves less biased information about the IM patients’ independence than for example the retrospectively estimated KPS values. In fact, we were able to determine who lived at home preoperatively and 3 months after surgery for 100% of the patients. Third, the reported series is the largest hospital-based series of operated very old IM patients to date.

Our study is not without limitations. Like previous studies, our study was retrospective in design, and thus pre- and postoperative assessments were not standardized. Therefore, we tried to use unambiguous measures for preoperative frailty (i.e. losing the CLH) as well as for the postoperative outcome (i.e. death and return to home). Moreover, as we had no information about the causes of deaths occurring after the hospital discharge, we could not determine if these deaths were related to surgery per se. On the other hand, we believe that future studies with prospective data collection methods are more suitable to evaluate the impact of a more detailed performance status, multiple comorbidities, minor postoperative complications and causes of death after surgery. As another limitation, since the survival rates of the general Finnish population are publicly available at the national level, we were not able to compare the observed survival rates to the region-matched survival rates of the general population of the HUH catchment area. In the most optimal situation, comparisons could have been made to conservatively treated very old IM patients with similar symptoms. However, such comparisons would perhaps be impractical or even unethical to conduct due to a poor prognosis of conservatively treated frail and very old IM patients. In fact, the reported mortality rates may overestimate the risks of surgery in comparison to a conservative treatment of symptomatic very old IM patients. Third, our cohort contained patients from a single, high-volume and academic institution, and therefore the results may have a limited external value. However, the results of excess mortality and return to home may serve as benchmark figures for future studies, which are surely needed as populations continue to age rapidly. Finally, many of the promising findings in our study may relate to the selection bias, i.e. patients have been carefully selected for surgery. In fact, nearly all (99%) operated patients were capable to live at home before the onset of meningioma-related symptoms, whereas 87% of the age-matched general population in the HUH catchment area lived at home between 2010 and 2018 (https://sotkanet.fi/sotkanet/en/index). In addition, as we had no information about patients who were conservatively treated, our results cannot be extrapolated to all very old IM patients. Nevertheless, one-third of the operated patients had a poor functional status and lost their CLH at the time of surgery. Therefore, at least some of the patients were rather fragile at the time of major surgery.

## Conclusions

Despite short-term excess mortality, very old patients operated for IM seem to have similar long-term survival rates as compared to the matched general population. As most operated patients sustained or regained their capability to live at home, surgery of selected, symptomatic and very old IM patients may be justified. Since preoperative dependency seems to increase the risk of adverse outcomes, surgical treatment could perhaps be considered at the very time when these IM patients become symptomatic.

## Supplementary Information

Supplementary Information.

## Data Availability

The datasets generated and analyzed during the study are not publicly available and the authors do not have permission to share the data. The access to used dataset and material needs to be requested from the local institutional review board of Helsinki University Hospital. More information can be inquired from the corresponding author.
