# Supplementary material for: Mortality of surgically treated 80-year-old or older intracranial meningioma patients in comparison to matched general population
Source: Sci Rep. 2021 Jun 1;11:11454. doi: 10.1038/s41598-021-90842-y (PMC8169827; doi:10.1038/s41598-021-90842-y)
Supplement: Supplementary file 1 — Supplementary Information. [file 41598_2021_90842_MOESM1_ESM.pdf]

Mortality of surgically treated 80-year-old or older intracranial meningioma patients in comparison to matched general population

Ilari Rautalin*, BM[1]; Christoph Schwartz, MD, MHBA[1,2] Mika Niemelä, MD, PhD[1]; Miikka Korja, MD, PhD[1]

[1]Department of Neurosurgery, University of Helsinki and Helsinki University Hospital, Helsinki, Finland

[2]Department of Neurosurgery, University Hospital Salzburg, Paracelsus Medical University, Salzburg, Austria

*Correspondence to Ilari Rautalin, Department of Neurosurgery, University of Helsinki, P.O. Box 266, FI-00029 Helsinki, Finland; E-mail address: ilari.rautalin@helsinki.fi; Telephone: +358 947187604 Fax: +358 947187616

Supplementary Files

Supplementary Table 1. Recorded major complications among the 83 operated very old IM patients.

Major complication, n (% of all operated patients)	
Any	22 (26.5)
Major intracranial bleeding*	8 (9.6)
New epileptic seizure**	5 (6.0)
Pneumonia	5 (6.0)
DVT/PE/sinus thrombosis	4 (4.8)
Postoperative ischemic lesion	3 (3.6)
New hemiparesis	2 (2.4)
Oculomotor paresis	2 (2.4)
Cardiac arrest	1 (1.2)
Hydrocephalus	1 (1.2)
CSF leakage requiring new craniotomy	1 (1.2)

*we considered postoperative intracranial bleeding as a major complication if it caused mass effect or required new craniotomy or trepanation

**we considered epileptic seizure as a major complication if both convulsions and a loss of consciousness were reported to medical notes. Recorded absence seizure was considered as a minor complication

CSF=cerebrospinal fluid; DVT=deep venous thrombosis; PE=pulmonary embolism

Supplementary Table 2: Odds ratios (ORs) with 95% confidence intervals (95% CIs) for major postoperative complication. Multivariate model includes significant factors from univariate model and patients' sex.

Variables	Major postoperative complication, OR (95% CI)	
	Univariable	Multivariable
Age (per each year)	1.32 (1.07-1.61)	1.32 (1.08-1.61)
Sex		
Male	(Reference)	(Reference)
Female	0.97 (0.34-2.76)	1.18 (0.37-3.73)
Helsinki ASA score		
2-3	(Reference)	NA
4	1.65 (0.62-4.42)	NA
Preoperative KPS (per 10-unit increase)	0.84 (0.62-1.14)	NA
Loss of capability to live at home preoperatively		
No	(Reference)	NA
Yes	1.37 (0.49-3.82)	NA
Tumor size (per one cm increase)	1.15 (0.82-1.61)	NA
Tumor location		
Other	(Reference)	NA
Skull-base	1.77 (0.66-4.75)	NA
Surgical time (per 30 min increase)	1.06 (0.89-1.25)	NA
Extent of resection		

Partial	(Reference)	NA
Total	(omitted)	NA

ASA= American Society of Anesthesiologist; KPS = Karnofsky Performance Status; NA = not applicable
